# Cobalt-induced neuro-behavioural alterations are accompanied by profound Purkinje cell and gut-associated responses in rats

**DOI:** 10.5620/eaht.2023010

**Published:** 2023-05-17

**Authors:** Akinleye Akinrinde, Kabirat Adigun, Oluwaseun Mustapha

**Affiliations:** 1 Department of Veterinary Physiology and Biochemistry, Faculty of Veterinary Medicine, University of Ibadan, Ibadan, Oyo State, Nigeria; 2 Department of Veterinary Anatomy, College of Veterinary Medicine, Federal University of Agriculture, Abeokuta, Ogun State, Nigeria

**Keywords:** Cobalt, cognition, memory, microbiota-gut-brain axis, vagus nerve

## Abstract

Metal ions including cobalt (Co) ions reportedly exhibit neurotoxic and antimicrobial properties. We hypothesized that oral exposure to Co may have implications for gut-dysbiosis with possible alterations of microbiota-gut-brain signaling in the host. In this preliminary study, we sought to examine whether exposure of male Wistar rats to cobalt chloride (CoCl_2_) at 0, 25, 50 and 100 mg/kg for two weeks affects select neurobehavioural indices, vagus nerve and brain morphology along with evaluation of associated changes in faecal bacterial flora, faecal fatty acids and the morphology of the intestines. CoCl_2_-exposed rats showed a dose-dependent reduction in hanging latency in the hanging wire (HW) test, reduced tendency to recognize novel objects in a Novel Object recognition (NOR) test, but increased interaction with open arms in the elevated plus maze (EPM) test, compared to controls. There were dose-dependent reductions in total heterotrophic count, coliforms, *E. coli, Enterococcal* and *Lactobacilli* counts in the faeces. Administration of CoCl_2_ at 100 mg/kg evoked the appearance of unsaturated fatty acids including palmitoleic, oleic and linoleic acids in the faeces as detected by gas chromatography-flame ion detection (GD-FID) analysis using fatty acid methyl esters (FAME) standards. Histopathological examination revealed chromatolysis of Purkinje cells in the cerebellum, although no significant lesions were present in the vagus nerve isolated from all the groups. In the intestines, there was moderate to severe infiltration of inflammatory cells into the duodenum, ileum, jejunum and colon while villi erosions were seen prominently in the ileum. These initial findings suggest that short-term exposure to Co can lead to gut-associated changes that may underlie neurotoxicity and alterations in behavior induced by Co.

## Introduction

The influence of cobalt (Co) and other lesser-studied environmental chemicals on neurological functions and behavior has gained increasing attention due to their use in a wide range of applications [1]. Exposure to Co has been associated with neurotoxicity in both *in vivo* and *in vitro* studies [2, 3, 4]. Among other possibilities, the main underlying mechanism of Co-induced neurotoxicity is believed to be the overproduction of free radicals and induction of oxidative stress when portions of absorbed cobalt circulate to and accumulates in the brain tissues [5]. However, in many cases, high concentrations of unabsorbed heavy metal ions remain in the gastrointestinal tract for considerable periods where they may directly impact on gastrointestinal structures and the gut microbiota which are essential for the overall physiology and maintenance of the health of the host. Alterations to gut microbial composition (dysbiosis) and structural integrity of the mucosal barrier are known to play important roles in bi-directional communications between the brain and the gastrointestinal tract, in what is commonly referred to as the microbiota-gut-brain axis [6, 7].

Previous pharmacokinetic studies of orally administered Co in rats indicated that the bulk (about 74.5%) of an oral dose of Co was eliminated in faeces, after about 36 hours of administration, with the likelihood that this portion is retained long enough in the gastrointestinal tract to interact with gut components, including the microbiota. Furthermore, the major portion of the Co absorbed in the intestines is accumulated mainly in tissues such as the liver, heart and kidneys, with only few amounts reaching the brain [8]. In effect, direct neurotoxicity resulting from distribution of absorbed Co to the brain may only account for a small proportion of its reported neurotoxic effects, raising suggestions that indirect mechanisms involving gut-brain connections may be involved in the toxic process. The classical exposure routes to cobalt involving accidental or deliberate ingestion of Co in drinking water or food, over-the-counter cobalt supplements or its use in doping of athletes or race-horses appear more relevant in this regard [9].

Low-grade inflammation induced by chemicals (e.g. cobalt) in the gut often produce shifts in microbiota composition, increased gut permeability and subsequent leakage of bacteria and their potential inflammatory and neuroactive metabolites (e.g. fatty acids, leucine, uric acid, α-synuclein etc.) into blood circulation from where they can reach the brain [10]. Some metabolites may also undergo prion-like translocation from the enteric nervous system to the CNS via the vagus nerve [11]. The vagus nerve is a major source of afferent information regarding visceral states and there are strong evidences from animal studies that activation of the vagus nerve by gut microbes plays critical roles in mediating effects on the brain and behavior [12]. Therefore, Co-induced intestinal inflammation progressing into a more generalized inflammatory response arising from perturbations of gut homeostasis may provide an important contribution to neurotoxic symptoms observed during Co exposure.

Potential disruptions of microbiome-gut-brain axis have been reported for relatively well-investigated metals such as cadmium [13], arsenic [14], chromium and nickel [15], occurring via microbial and structural alterations in gut homeostasis [16, 17]. However, studies describing the perturbations in the composition, structure, diversity and metabolic activity of gastrointestinal bacteria following exposure to Co are still very rare. To further understand potential relationships between the gut and brain during cobalt exposure, the present study was designed to examine Co-induced alterations in memory and learning, anxiety behavior and muscular coordination in rats and how these changes reflect in or correlate with some components of gut integrity including faecal bacteria and fatty acid profiles as well as the morphology of different segments of the intestines, brain and vagus nerve.

## Materials and Methods

### Animals and Experimental Groups

Forty-eight male Wistar rats, obtained from the animal facility of the Experimental Animal Unit, Faculty of Veterinary Medicine, University of Ibadan, were randomly allocated into four groups labeled A-D (n = 12 per group). The rats were housed in plastic cages in a well-ventilated animal house with room temperature between 26 and 29ºC and a 12 h light: 12 h dark photoperiod daily. Standard rat chow and clean drinking water were provided for the rats throughout the duration of the experiment including an initial one-week period for acclimatization. The conduct of the experiments followed guidelines contained in the “Guide for the Care and Use of Laboratory Animals” [18], published by the National Institute of Health and were guided by protocols approved by the institutional Animal Ethics Committee

The dosages of Co used in this study were chosen based on previous toxicity reports by different authors [15, 19, 20]. Group A received normal saline and served as the control group. Rats in Groups B, C and D were given CoCl_2_ at 25, 50 and 100 mg/kg by oral gavage in daily single doses for 14 consecutive days. Rats were weighed immediately prior to commencement of dosing with CoCl_2_ and prior to termination of the experiment on the 15th day.

### Neurobehavioural tests

#### Hanging wire test

On the 14th day, few hours after the last dosing, motor coordination and balance in the experimental rats were assessed by evaluating their hanging latency on a piece of steel wire (about 80 cm in length and 2 mm in diameter) secured horizontally at both ends at a height of about 1 m above the ground level. The conduct of this test followed procedures previously described by Van Putten *et al.* [21]. Briefly, the rats were suspended, one at a time, with their forepaws gripping the piece of steel wire. Once an animal grips the wire with the forepaws, it is released and allowed to hang vertically. The time it takes for the rat to fall (latency to fall) was recorded using a stopwatch and the trial was conducted twice for each rat, with a 6 h interval between trials.

#### Novel object recognition

The test was carried out as described by Lueptow [22]. It was used to assess rats’ cognitive abilities following exposure to Co, as rats are believed to prefer exploration of novel objects rather than familiar ones. Briefly, the test commenced on day 12 with habituation of rats by placing them in the centre of the test apparatus consisting of a wooden square chamber (about 40 cm x 40 cm x 40 cm) and allowing exploration for about 5 min. This was followed by training on day 13 where rats were placed in the middle of the box and allowed to interact with two identical objects placed at opposite ends of the apparatus for about 10 min. On testing day (day 14), one of the initial objects (familiar object) was placed at one end of the arena, while a novel object was placed in the opposite end of the apparatus. Each rat was placed in the center of the arena and allowed to freely explore the arena for about 10 min. The time spent in the centre of the arena was recorded, while the discrimination index was calculated as the time spent exploring the novel object minus the time spent exploring the familiar object divided by total exploration time. Values of discrimination index are normally expected to fall between -1 and +1.

#### Elevated Plus Maze

The plus maze apparatus included a central platform (5cm x 5 cm) from which emerged two open arms each of equal dimensions (length: 30 cm; width: 5 cm; height: 0.25 cm) and two closed arms of equal dimensions (length: 30 cm; width: 5 cm; height: 15 cm). The two identical arms are placed opposite each other from the central platform, while the apparatus was elevated at about 40 cm from the ground. On testing day (day 13), rats were selected randomly and initially placed on the central platform, while facing one of the open arms and then allowed to move freely for standard 5 min duration, while recordings of classical behavioural parameters of anxiety in the maze were made by a trained observer who was blinded to the treatments administered. The maze was cleaned thoroughly with methylated spirit after each session with each rat. The parameters collected included: (i) Frequency in open arms, which describes the number of entries of a rat with all four paws into the open arms, (ii) Duration in open arms defined as the total amount of time spent by the rat in the open arms, (iii) Frequency in closed arms, indicating the frequency of rat entry into the closed arm, and (iv) Duration in closed arms, describing the total amount of time spent by each rat in the closed arms.

### Gas Chromatography-Flame Ion Detection (GC-FID) analysis of faecal fatty acids

The extraction of fatty acids from faecal samples was carried out according to methods described by Scortichini *et al.* [23], with slight modifications. Faecal samples collected fresh from voiding were initially frozen at -20ºC until the analysis was carried out. Briefly, the samples were first homogenized thoroughly and about 100 mg was weighed into a vial and then acidified with sulphuric acid (0.25 mL, 50 % w/v). The solution was mixed using a vortexing device for about 3 min. Thereafter, fatty acids were extracted by adding 1 mL of diethyl ether followed by centrifugation at 2,800 g for 5 min. The procedure was repeated three times for complete extraction into the solvent. About 0.5 µ L of the collected organic phase was then injected into the GC column calibrated with fatty acid methyl ester (FAME) mix standards for the analysis of medium- and long-chain fatty acids.

Fatty acid methyl ester was separated by Perkin Elmer capillary Gas Liquid Chromatography comprising a flame ionization detector and a capillary column (RT-2560, 50 m x 0.25 mm I.D, 0.25 micron dry film) housed in a temperature regulated oven. Temperature programming was observed thus: the initial oven temperature was 70 ºC, held for 5 minutes, and then rose to 200 ºC at a rate of 4 ºC/min held for 0 minute. It was further raised to 202 ºC for 2 minutes at 0.1 ºC/min held for 0 minute.

The injector and detector temperatures were set at 250 ºC and 280 ºC, respectively. The carrier gas was nitrogen at a flow rate of 30 psi. The injection volume was 1 µ L in a splitless mode and the sample’s run time was 57.50 minutes. Fatty acids were identified by comparing the retention times of FAME with a standard 37 component FAME mixture (Restek). The components eluting from the column were detected by flame ionization detector whose signal output was captured and recorded in computer with Totalchrom software data system.

### Histopathology of the brain, intestines and vagus nerve

The rats were sacrificed and perfused transcardially first with normal saline followed by 10% buffered formalin. Thereafter, the brain was dissected from each rat and the vagus nerve was carefully isolated from the cervical region by a trained anatomist. The intestines were removed and small portions of the duodenum, jejunum, ileum and colon were dissected. All the isolated tissues were immediately transferred into 10% formalin and later embedded in paraffin. Sections of about 5 µ m thickness were made with a rotary microtome and they were prepared for staining with haematoxylin and eosin (H&E) by conventional methods [24]. Specifically, the selected brain regions of interest were the hippocampus, prefrontal cortex and cerebellum. The tissue slides were then observed under an Olympus® light microscope while photomicrographs were taken with a digital camera.

### Determination of viable faecal bacterial count

Fresh faeces were collected aseptically from the different groups of rats prior to their euthanasia and the enumeration of bacteria from freshly voided, serially diluted faecal pellets was carried out on different selective media, according to the pour plate technique described by Harrigan and MacCance [25]. Culture media were obtained from Oxoid Ltd, Basingstoke, United Kingdom and included MacConkey agar (coliforms), Eosin methylene Blue agar (*Escherichia coli*), Slanetz and Bartley medium (*Enterococcus spp.*) and Mannitol salt agar (*Staphylococcus spp.*), De Man Rogosa and Sharpe (MRS) agar (*Lactobacilli*) and Centrimide agar (*Pseudomonas aeruginosa*). Bacteria were enumerated as colony forming units per gram of faeces (CFU/g).

### Statistical analysis

Data were analyzed using GraphPad Prism software (version 7.00). The data were expressed as mean±standard deviation. Behavioural data, body and brain weights and the quantification of bacterial populations were analyzed by One-way analysis of variance (ANOVA) followed by Tukey’s post hoc test for multiple comparisons. P values less than 0.05 were considered statistically significant.

## Results

### Changes in body and brain weights

Body weight gain over the course of the experiment was highest (29.13%) in the control group while body weight gain decreased with increasing dose of CoCl_2_ (Table 1). Rats in Group D (100 mg/kg CoCl_2_) showed no apparent increase in weight after the experiment. Brain weights were not significantly different across the different groups.

### Behavioural test results

#### Hanging wire test

**Figure 1.**
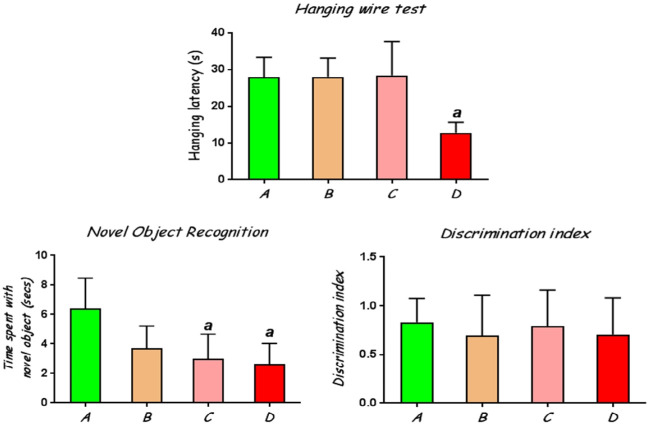
(A) Latency of fall in the hanging wire test, (B) Time spent with novel object in the centre of field in the Novel Object Recognition test and (C) The Discrimination index, a measure of memory sensitivity in the rats. A, control group; B, CoCl_2_25 mg/kg; CoCl_2_50 mg/kg; CoCl_2_100 mg/kg. a: significantly different from control group (p<0.05).

Rats treated with CoCl_2_ at 100 mg/kg had significantly (p<0.05) lower hanging latency on the wire compared to the control rats (Fig. 1A).

#### Novel Object Recognition

This test was employed to assess learning and memory in the experimental rats by evaluating their interaction with a novel object when exposed alongside a previously presented familiar object. The results showed that rats treated with CoCl_2_ at 50 mg/kg and 100 mg/kg had a significantly reduced tendency to distinguish between the novel and familiar objects in that they spent lesser times with the novel object compared with the control rats (Fig. 1B). However, all the groups of rats showed positive discrimination indices (Fig. 1C), indicating that they all retained memory of the familiar objects, although this was slightly higher in the control rats than the CoCl_2_-exposed rats.

#### Elevated plus maze

**Figure 2.**
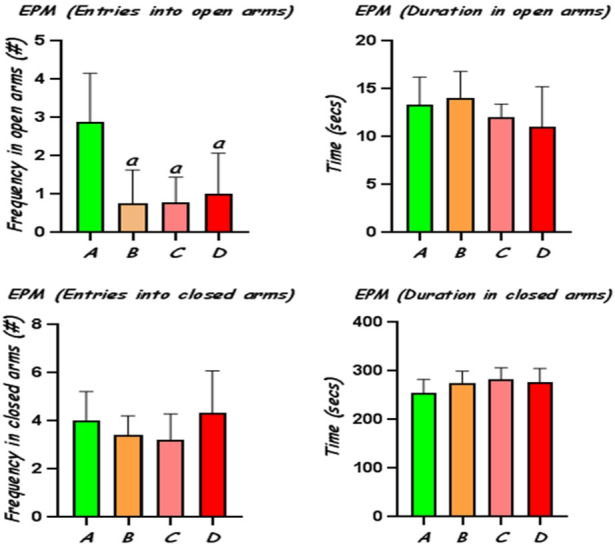
Frequency of entries and time spent in the open and closed arms of an elevated plus maze by rats treated with CoCl_2_. A, control group; B, CoCl_2_25 mg/kg; CoCl_2_50 mg/kg; CoCl_2_100 mg/kg; a: significantly different from control group (p<0.05).

Administration of CoCl_2_ at 25, 50 and 100 mg/kg elicited significant (p<0.05) reduction in open-arms exploration with decreased number of entries in the open arms, compared to the control, indicating increased anxiety behaviour in these rats (Fig 2). The frequency of entries into the closed arms and time spent in closed arms was not significantly altered in the different groups, although rats treated with CoCl_2_ showed increased tendency for interaction with the closed arms.

### Brain Histology

**Figure 3.**
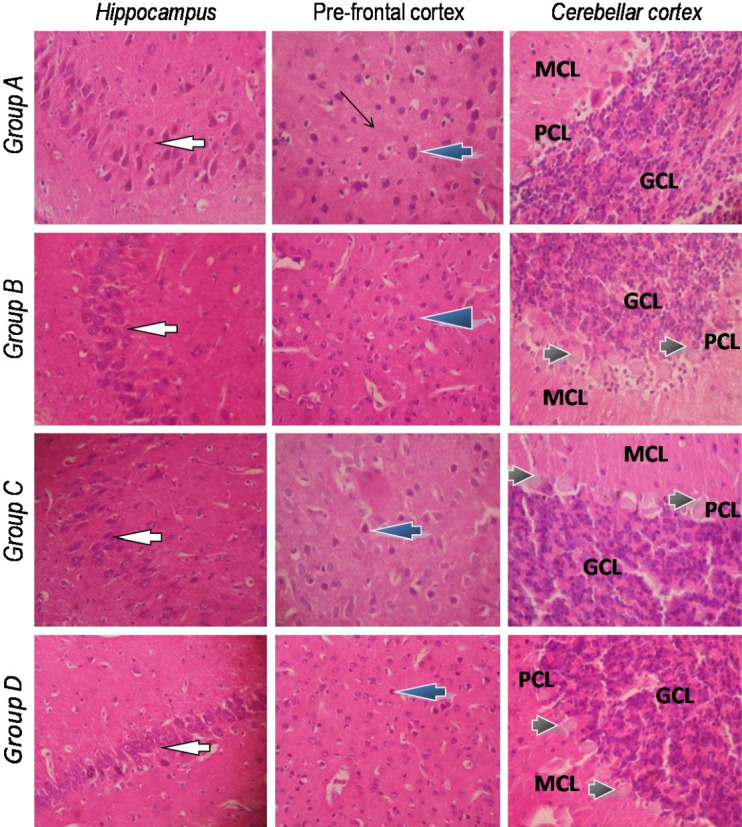
Photomicrographs of brain sections from the experimental rats showing the Hippocampus, prefrontal cortex and the layers of the cerebellum. Group A (control): normal organization of the different subfields of the hippocampus and normal neuronal cells (white arrow); pre-frontal cortex shows normal laminae, normal neuronal cells (blue arrow) and normal stroma (slender arrow); the cerebellar cortex shows normal folia, normal Molecular cell layer (MCL), Purkinje cell layer (PCL) and Granular cell layer (GCL). Group B (CoCl_2_ 25 mg/kg): normal hippocampus and prefrontal cortex as in Group A; The MCL and GCL appear normal, however, the PCL shows several chromatolysed purkinje cells (black arrows). Group C (CoCl_2_ 50 mg/kg): normal hippocampus and prefrontal cortex; the MCL and GCL also appear normal, but the PCL shows several chromatolysed purkinje cells (black arrows). Group D (CoCl_2_ 100 mg/kg): normal hippocampus and prefrontal cortex; The MCL and GCL of the cerebellum also appear normal, although, the PCL shows severe chromatolysis of purkinje cells (black arrows). (H&E; Magnification: x400).

Histological sections of the brain showing the hippocampus, prefrontal cortex and cerebellum are shown in Fig. 3. The sections from the various groups showed no significant lesions in the hippocampus which presented normal pyramidal neurons. The structural architecture of the hippocampal sub-regions: CA1, CA2 and CA3 also appear normal. Similarly, the prefrontal cortex showed normal laminae, neuronal cells and neuropil in all the different groups. The histological lesions seen in the brain, however, seemed to reside in the cerebellum. In control rats, the Purkinje cells located between the molecular and granular cell layers had distinct cell bodies and were well arranged. However, the cerebellum from all CoCl_2_-exposed rats presented Purkinje cells undergoing varying degrees of chromatolysis with slight disorientation in the Purkinje cell layer.

### Vagus nerve histology

**Figure 4.**
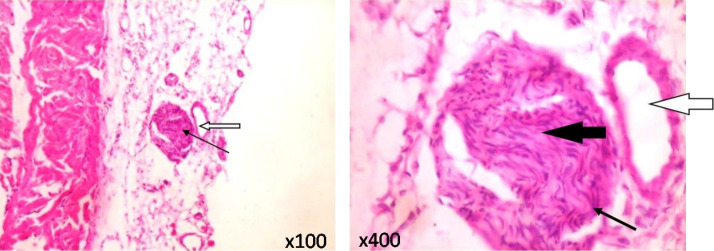
Typical morphology of the transverse section of the cervical vagus nerve isolated from CoCl_2_-treated rats at 100 mg/kg. Sections show normal perineurium and epineurium (slender arrow). The endoneurial compartment (black arrow) appears normal with nerve fibres and their accompanying Schwann cells, adjoined by a large epineurial blood vessel (white arrow). No morphological differences were observed between the vagus nerve from control rats (not shown) and the CoCl_2_-treated rats. (H&E; Magnification: x100; x400).

As depicted in Figure 4, typical vagus nerves isolated from rats in the control and CoCl_2_-treated groups showed normal neural architecture with normal perineurium, epineurium and endoneurial compartment.

### Intestinal histology

**Figure 5.**
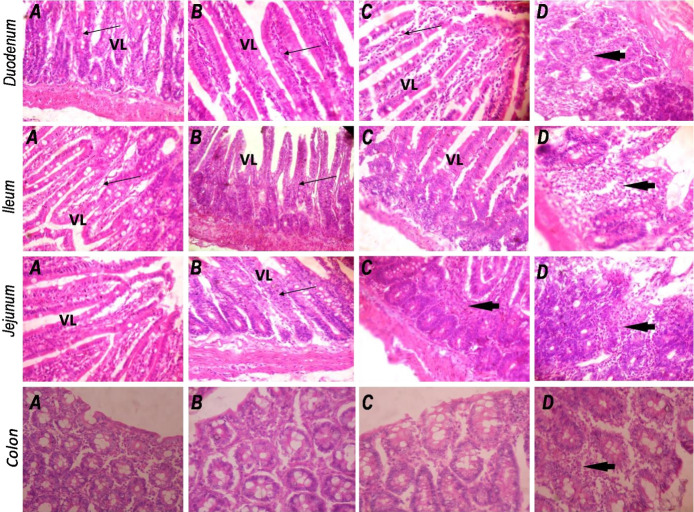
Morphology of the duodenum, jejunum, ileum and colon in the experimental rats. A, control group; B, CoCl_2_25 mg/kg; C, CoCl_2_50 mg/kg; D, CoCl_2_100 mg/kg. VL, villi. Group A (control): well-preserved mucosal layers in all intestinal segments with well-arranged villi in the duodenum, jejunum and ileum, normal lamina propria (slender arrow) and very mild or complete absence of inflammatory cells. Group B (CoCl_2_ 25 mg/kg): showed similar mucosal architecture to that of control rats. Group C (CoCl_2_ 50 mg/kg): photomicrographs show moderately preserved epithelium, normal villi in the small intestine, but moderate infiltration of inflammatory cells in the ileum (black arrow). Group D (CoCl_2_ 100 mg/kg): the duodenal, jejunal, ileal and colonic mucosa were all poorly preserved, villi are poorly defined and there was moderate to severe infiltration of inflammatory cells. (H&E; Magnification: x400).

Sections of the duodenum, ileum, jejunum and colon were examined for associated changes in the intestinal architecture following CoCl_2_ exposure. In all segments of the intestine, no histological differences were noted between the control group and rats treated with 25 mg/kg CoCl_2_ (Fig. 5). The intestinal mucosa in these groups showed normal, well preserved epithelium with well-arranged villi in the different small intestinal segments examined. However, treatment with CoCl_2_ at 50 and 100 mg/kg provoked moderate to severe infiltration of inflammatory cells which include lymphocytes, polymorphs and plasma cells in the lamina propria and submucosa of the duodenum, ileum, jejunum and colon. Very importantly, CoCl_2_ administered at 100 mg/kg produced severely inflamed mucosal layers with eroded and indistinct villi in the duodenum, jejunum and ileum. The colonic mucosa also showed moderate infiltration of inflammatory cells in the rats exposed to 100 mg/kg CoCl_2_.

### Faecal fatty acid analysis

Recent studies have suggested the use of faecal fatty acids (short- and long-chain) as a novel screening tool for disorders of the gut, including colorectal cancers. Here, we employed a mixture of C8 – C24 fatty acid methyl esters (FAME), analytical standards to quantify levels of fatty acids in the faeces of rats exposed to increasing levels of CoCl_2_ using GC-FID as described by Scortichini et al [23]. The results showed the presence of medium- and long-chain fatty acids ranging from C8 to C20 (Table 2). Regarding the composition of fatty acids, the highest variability of fatty acids was found in faecal samples from rats treated with CoCl_2_ at 100 mg/kg, including saturated fatty acids (C:8, C11:0, C13:0 and C16:0), and monounsaturated fatty acids (C16:1, C18:1n9c and C18:2n6c). Treatment with CoCl_2_ at the lower doses (25 and 50 mg/kg) did not produce considerable alterations in faecal fatty acid profiles compared to the control. Undecylic acid (C11:0) and palmitic acid (C16:0) were the only fatty acids common to faecal samples from all the experimental groups, while arachidic acid (C20:0) was detected only in rats treated with CoCl_2_ at 25 mg/kg. The most abundant fatty acids detected in the different groups were myristic acid (67.02%), arachidic acid (45.68%), myristic acid (44.00%) and tridecyclic acid (51.66%) for 0, 25, 50 and 100 mg/kg CoCl_2_, respectively.

### Faecal bacterial flora

**Figure 6.**
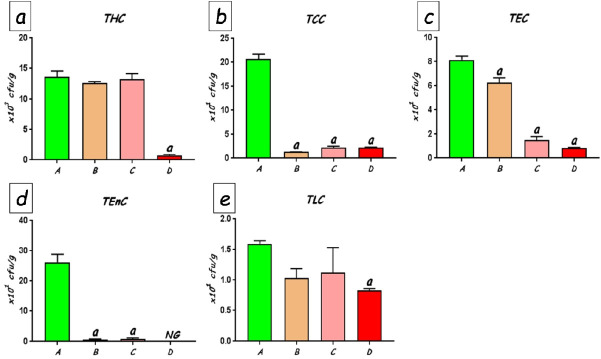
Culture counts of faecal bacteria in rats exposed to CoCl_2_. A, control group; B, CoCl_2_25 mg/kg; C, CoCl_2_50 mg/kg; D, CoCl_2_100 mg/kg. THC, Total heterotrophic count; TCC, Total coliform count; TEC, Total E. coli count; TEnC, Total Enterococci count; TLC, Total lactobacilli count. NG, No growth; cfu, colony forming units per gram of faeces. a Significant compared to Group A (p<0.0001).

The total counts of faecal bacteria enumerated on different selective media are shown in Figure 6. No significant differences were found in the total numbers of viable bacteria between the control rats and the rats treated with 25 and 50 mg/kg of CoCl_2_. Culture counts in these groups averaged between 12.6 x 10^3^ and 13.6 x10^3^ colony forming units (cfu) per gram of faeces (Fig. 6a). However, treatment with CoCl_2_ at 100 mg/kg produced significant (p<0.0001) reduction total viable bacteria (to an average of 0.76 x10^3^cfuper gram faeces) compared with the other groups. In similar fashion, culture counts of various groups of bacteria were significantly reduced following CoCl_2_ exposure. Coliform counts which averaged 20.63 x 101cfu per gram of faeces was drastically reduced to as low as 1.38 x10^1^cfu per gram of faeces (p<0.0001). The faecal counts of Escherichia coli (Fig. 6c), Enterococcal (Fig. 6d) and lactobacilli (Fig. 6e) counts also showed significant dose-dependent reductions when compared with the control rats, with the highest dose of CoCl_2_ producing the greatest decline in bacterial populations. Pseudomonas and Staphylococcus species were, however, undetected in the faecal samples from all the rats.

## Discussion

The goals of the current study were to determine whether neurobehavioural alterations following sub-acute (14-day) exposure to increasing doses of cobalt chloride may be associated with changes in selected faecal bacteria populations, fatty acid profiles and histopathological changes in the gastrointestinal tract, vagus nerve and brain. Heavy metals including Co are known to promote neurotoxicity via increased production of reactive oxygen/nitrogen species and the induction of oxidative stress in nervous tissues [26]. However, this is thought to only provide a partial explanation for the nature of neurobehavioural alterations that accompany exposure to Co [27]. In the present study, treatment of rats with CoCl_2_ for 14 days produced a dose-dependent reduction in body weight gain, while brain weights were unaffected. The reduction in body weight could be attributed to changes in feeding and drinking habits, notably a reduction in appetite observed in the Co-treated rats. Previous reports have indicated that some heavy metals such as Pb, Cd, Cu, Hg possess the ability to interfere with the regulation of appetite via the central nervous system [28, 29].

In the present study, assessment of motor coordination in a simple hanging wire test showed a sharp decrease in hanging latency when exposed to CoCl_2_ at 100 mg/kg. This result is consistent with our previous study [3] that utilized a higher dosage of cobalt than the ones used in the present study. Co is known to interfere with synaptic transmission via blockage of pre-synaptic calcium channels and reduction of neurotransmitter-induced post-synaptic responses [30] and this may be responsible for the loss of motor coordination observed in Co-exposed rats. However, Oria *et al.* [31] reported significant reduction in the hanging latency of rats exposed to CoCl_2_ at a lesser dosage of 40 mg/kg administered for 14 days.

Along with the loss of motor coordination, Co-treated rats showed progressive impairment of memory and learning with increasing dosage of CoCl_2_ in the Novel object recognition (NOR) test, as indicated by significantly reduced times spent with a novel object placed in the centre of a field. The NOR test provides an opportunity to investigate learning and memory when animals are simultaneously presented with one new and one old object [32]. Following initial training with exposure to two identical objects, one of the objects becomes replaced with a new one. The object recognition memory is indicated by the length of time the animal spends with the novel object, having consideration for the fact that rats tend to demonstrate preference for exploration of novel, rather than familiar objects [22]. Studies on Co effects on memory and learning are rare, but few studies have reported significant memory loss and impaired attention in hard metal workers exposed to Co compared to unexposed subjects [33].

Furthermore, we employed an elevated plus maze (EPM) for evaluation of anxiety-related behaviour in the experimental rats. The EPM makes use of a wooden maze apparatus constructed with four arms in a plus shape, including two open and two closed arms all meeting at a junction in the centre of the apparatus [34]. For each trial, the rats were placed, one at a time, in the centre of the maze and the number of entries into the arms and duration of exploration of the arms were recorded by an independent observer. An increase in the activity of the rats in the open arms reflects lack of anxiety. The results from the present experiment revealed that rats exposed to Co displayed anxiety-related behaviour by significantly reduced entries into the open arms. Nevertheless, the exploratory activities of the rats in the closed arms were not significantly different compared to those of control rats. Our results suggest that Co may induce anxiogenic effects as supported by findings from previous studies using the Open Field Test (OFT) in rats [3, 31]. It is worth noting, however, that in some other models such as rats subjected to Vogel conflict test (VCT) and contextual fear conditioning, Co was found in a study to also exert anxiolytic-like effects in rats, although this was found to be reversible [35]. The conflicting evidence regarding the effects of Co on anxiety-related behaviour of rats may be a result of differences in the nature of the different models. While other behavioural assays of anxiety rely on the production of a conditioned response following presentation of noxious stimuli, the EPM produces unconditioned responses relying upon the natural inclination of rodents to seek out dark, enclosed spaces and avoid open spaces [34]

Histological examination of selected brain regions revealed that the hippocampus and pre-frontal cortex showed normal folia, stroma and cellular morphology. This may be due to the fact that these parts of the rat brain accumulate much lower amounts of Co than other tissues as indicated in a previous study [36]. However, the cerebellum and particularly the Purkinje cells appeared to be more susceptible to the toxic effect of Co. Purkinje cells, typically arranged in a single row of cells between the granular and molecular cell layers, are especially susceptible to toxic stress [37, 38]. The chromatolysis and degeneration of purkinje cells seen in Co-exposed rats are in congruence with findings in the brain of goats with history of heavy metal exposure from artisanal mining [39] and cerebellum of cadmium-exposed rats [40]. Necrosis, degeneration and chromatolysis of purkinje cells during toxicant exposure has been attributed to the induction of oxidative stress with the purkinje cells often the most sensitive to such stress [41]. Perhaps, the alterations in the histomorphology of the cerebellum, and particularly the Purkinje cells may be responsible for the loss of motor coordination as well as interference with learning and memory as obtained in this study [42, 43]

The vagus nerve (VN) serves as an essential part of the parasympathetic nervous system which senses gut information including the presence of microbiota metabolites and transmits to the central nervous system via its afferent fibres. The VN thus serve vital connectivity functions between the gut and brain and tends to inhibit peripheral inflammation via what is described as the cholinergic anti-inflammatory pathway [44]. Stressful conditions of the gastrointestinal tract and microbiota are known to inhibit VN function, resulting in inflammatory and irritable bowel diseases [45]. In the present study we sought to examine if Co exposure and resultant stress produced any changes in the morphology of the VN. We found that exposure to CoCl_2_ at the doses tested did not produce any significant changes in the morphology of the cervical VN isolated from rats in the various groups. Further, our examination of the various segments of the intestines revealed poorly preserved mucosa layers of the duodenum, jejunum, ileum and colon in Co-exposed rats, especially those treated with the highest dose (100 mg/kg). This damage to the intestinal mucosa was accompanied with moderate to severe degrees of inflammatory cell infiltration. It is reasonable to suggest that while Co caused extensive damage to the intestinal epithelium, these changes might have affected the biochemical components of the VN function, while the morphology remained unaffected. Extensive intestinal damage and inflammation as observed in Co-treated rats in this study has implications for increased intestinal permeability and transfer of gut microbiota and metabolites into the blood circulation from where they can reach the brain to affect its functions [46]. The involvement of specific gut- and microbiota-derived metabolites in this process will be the focus of further studies.

Inflammation in the intestines and the pathogenesis of inflammatory bowel disease has been linked to an abundance of saturated long-chain fatty acids, particularly n-6 long-chain, rather than n-3 polyunsaturated fatty acids [47, 48]. In this study, therefore, we carried out a metabolomic profiling of medium-chain and long-chain fatty acids in the faeces of Co-treated rats using GC-FID in order to assess its relationship to the extent of inflammation in the intestines. We found that the rats treated with CoCl_2_ at 100 mg/kg showed an increase in the diversity of LCFAs compared with the control rats and those receiving lower doses of CoCl_2_. Palmitoleic, oleic and linoleic acids were only found in the faeces of rats in the 100 mg/kg CoCl_2_ group, but not in the other groups. The results bear a direct relationship to the degree of inflammation observed in the intestines and suggest that LCFAs present in the intestines may play a role in the development of intestinal inflammation, and by extension, the remote effects in other tissues, including the brain. Generally, pro-inflammatory effects are often associated with n-6 PUFAs such as linoleic acid which can be metabolized to arachidonic acid with subsequent metabolism via the cyclooxygenase pathway resulting in the formation of inflammatory mediators such as prostaglandins, leukotreines and thromboxanes [49].

Heavy metal ions possess the ability to interact with vital structural components of the gastrointestinal tract, including the gut microbiota and also interfere with vital cellular functions eventually leading to cell death. In some cases, metals have been employed as pre-antibiotics or alternative antimicrobials capable of antibacterial, antifungal and antiviral activities [50]. Therefore, the potential to alter the homeostasis of several groups of bacteria in the intestinal flora is very high. In the present study, we observed a general decline in the total viable counts of groups of bacteria in the faeces of rats exposed to CoCl_2_, which in many cases were dose-dependent. While this antimicrobial effect may be beneficial in some cases, such as in the elimination of pathogenic or potentially pathogenic organisms, the indiscriminate nature of the activity of these metals may cause removal of commensal bacteria of the normal gut flora posing undesirable consequences and stimulating adverse remote events in other organs [51, 52]. Previous work by Richardson *et al.* [15] indicated significant shifts in gut microbiome when rats were exposed to Co or other toxic metals, but these were not related to alterations in behaviour or brain morphology in the rats. Future work will seek to understand the specific shifts in gut microbiota, metabolites and/or gut-related molecular pathways responsible for the neurobehavioural alterations using real-time PCR analysis to screen bacterial groups as well as 16S rRNA sequencing of the gut microbiome.

In conclusion, the current findings indicate that sub-acute exposure to low and medium doses of CoCl_2_ can result in loss of motor coordination, anxiety-like behaviour and impairment of memory, with effects more noticeable in the highest dose (100 mg/kg CoCl_2_) group. The different doses of CoCl_2_ exhibited antimicrobial activity by reducing the populations of viable bacteria in the gut, an effect that may likely have profound ramifications for gut-brain communications. Profound inflammatory responses and abundance of pro-inflammatory long-chain fatty acids in the intestines also provide evidence of Co-induced disruption of gut integrity which correlated with morphological changes observed in Purkinje cells in the cerebellum. With recent increase in deliberate and inadvertent Co consumption from various sources, the findings from the current study strongly suggest that pathophysiological links between the gut and brain are likely to be important contributors to neurotoxicity observed during Co exposure. However, future studies are required before firm and specific conclusions can be drawn in this regard.

## Figures and Tables

**Table 1.** The effect of sub-acute (14-day) cobalt chloride exposure on body weights and brain weights of rats

Groups	Body weight (g)		% Body weight change	Brain weight (g)
	Day 0	Day 15		
A	119.00±11.05	167.91±20.30^*^	+29.13	1.56±0.07
B	110.00±11.89	141.46±18.76^*^	+22.23	1.48±0.14
C	119.25±15.45	124.09±19.10	+3.90	1.48±0.05
D	122.17±16.83	122.00±13.42	-0.14	1.68±0.07

* indicates significant difference between initial and final weights in each group

**Table 2.** The effect of sub-acute (14-day) cobalt chloride exposure on composition of medium and long-chain fatty acids in the faeces of experimental rats

Fatty acid nomenclature	Component name	Area (%)			
		Control (0 mg/kg)	CoCl_2_ (25 mg/kg)	CoCl_2_ (50 mg/kg)	CoCl_2_ (100 mg/kg)
C8:0	Caprylic acid	5.06	-	-	3.10
C11:0	Undecylic acid	10.27	8.64	30.67	11.38
C13:0	Tridecylic acid	-	35.11	-	51.66
C14:0	Myristic acid	67.02	-	44.00	-
C16:0	Palmitic acid	17.65	10.58	25.34	16.45
C16:1	Palmitoleic acid	-	-	-	5.91
C18:1n9c	Oleic acid	-	-	-	5.79
C18:2n6c	Linoleic acid	-	-	-	5.71
C:20	Arachidic acid	-	45.68	-	-
Total		100	100	100	100
